# Perceptual-Cognitive Changes During Motor Learning: The Influence of Mental and Physical Practice on Mental Representation, Gaze Behavior, and Performance of a Complex Action

**DOI:** 10.3389/fpsyg.2015.01981

**Published:** 2016-01-08

**Authors:** Cornelia Frank, William M. Land, Thomas Schack

**Affiliations:** ^1^Neurocognition and Action-Biomechanics Research Group, Bielefeld UniversityBielefeld, Germany; ^2^Cognitive Interaction Technology – Center of Excellence (CITEC), Bielefeld UniversityBielefeld, Germany; ^3^Department of Kinesiology, Health, and Nutrition, University of Texas at San Antonio, San AntonioTX, USA; ^4^Research Institute for Cognition and Robotics (CoR-Lab), Bielefeld UniversityBielefeld, Germany

**Keywords:** motor imagery, skill representation, SDA-M, eye-tracking, quiet eye, golf putting, skill acquisition

## Abstract

Despite the wealth of research on differences between experts and novices with respect to their perceptual-cognitive background (e.g., mental representations, gaze behavior), little is known about the change of these perceptual-cognitive components over the course of motor learning. In the present study, changes in one’s mental representation, quiet eye behavior, and outcome performance were examined over the course of skill acquisition as it related to physical and mental practice. Novices (*N* = 45) were assigned to one of three conditions: physical practice, combined physical plus mental practice, and no practice. Participants in the practice groups trained on a golf putting task over the course of 3 days, either by repeatedly executing the putt, or by both executing and imaging the putt. Findings revealed improvements in putting performance across both practice conditions. Regarding the perceptual-cognitive changes, participants practicing mentally and physically revealed longer quiet eye durations as well as more elaborate representation structures in comparison to the control group, while this was not the case for participants who underwent physical practice only. Thus, in the present study, combined mental and physical practice led to both formation of mental representations in long-term memory and longer quiet eye durations. Interestingly, the length of the quiet eye directly related to the degree of elaborateness of the underlying mental representation, supporting the notion that the quiet eye reflects cognitive processing. This study is the first to show that the quiet eye becomes longer in novices practicing a motor action. Moreover, the findings of the present study suggest that perceptual and cognitive adaptations co-occur over the course of motor learning.

## Introduction

Research in sports has shown that experts do not only differ from novices in their reproducibly superior performance, but also in their perceptual-cognitive background, i.e., in their underlying skill representation (e.g., Ericsson and Smith, 1991; Schack and Mechsner, 2006; Ericsson, 2007; Hill, 2007) as well as in their gaze behavior (e.g., Vickers, 1992; Williams et al., 2002; Campbell and Moran, 2014). To this extent, perceptual-cognitive abilities are seen as one of the key components associated with high-level performance. With respect to skill acquisition, recent research has indicated that practice has a significant influence on the cognitive system underlying motor learning and performance (e.g., Frank et al., 2013; Land et al., 2014). Specifically, practice has been shown to lead to the establishment of mental representations, which are stated to aid motor performance (e.g., Ericsson, 2003; Land et al., 2013). Moreover, different types of practice have been shown to differentially influence mental representation development, with practice that incorporates motor imagery and practice that focuses on movement effects adding to the development of mental representations of the action (e.g., Frank et al., 2014; Land et al., 2014). However, in an attempt to provide a more holistic account of the changes associated with learning, and the influence of different learning strategies, it is important to consider associated perceptual changes accompanying those seen in both the cognitive and motor system.

The most common means to acquire a motor skill and to induce persistent improvement in performance is through physical repetition of the overt movement to be learned. However, research has also shown that mental practice, either alone or combined with physical practice, can also aid in the acquisition of skill. This form of practice is based on mental repetition by way of motor imagery that is the covert simulation of a movement in one’s mind without subsequent movement execution (e.g., Jeannerod, 2001, 2004; Moran et al., 2012). While both types of practice have shown to influence performance and to promote motor learning (e.g., Feltz and Landers, 1983; Feltz et al., 1988; Hinshaw, 1991; Grouios, 1992; Driskell et al., 1994), physical practice has been demonstrated to be superior to mental practice, but mental practice has been shown to be better than no practice at all (for a meta-analysis, see Driskell et al., 1994). Apart from the obvious changes in motor performance associated with these practice strategies, the perceptual-cognitive changes that occur with respect to mental or physical practice are less clear.

Gaze behavior, and in particular the quiet eye phenomenon, has been considered a key element in the perceptual-cognitive processes underlying learning and performance. According to Vickers (2009, p. 280), the quiet eye is defined as

“the final fixation or tracking gaze that is located on a specific location or object in the visuomotor workspace within 3° of visual angle for a minimum of 100 ms. The onset of the quiet eye occurs prior to the final movement in the task and the offset occurs when the gaze deviates off the object or location by more than 3° of visual angle for a minimum of 100 ms (…).”

This behavior (i.e., the final fixation prior to movement onset, such as the onset of the backswing during golf putting) is thought to be an important indicator of action-related information processing. The quite eye phenomenon has been observed and investigated across a variety of sports and motor tasks (for reviews, see Vickers, 2007, 2009, 2011), including golf (e.g., Vickers, 1992; Wilson and Pearcy, 2009; Vine and Wilson, 2010; Vine et al., 2011; Wood et al., 2013; Ziv and Lidor, 2015).

Up to now, research on quiet eye has elicited distinct differences in this type of gaze behavior between skilled and non-skilled performers. Particularly, and probably the most prominent finding so far, is that the quiet eye duration of skilled performers is longer compared to non-skilled performers (e.g., Vickers, 1992, 1996; Janelle et al., 2000; Williams et al., 2002). Moreover, skilled performers have been shown to perform with an optimal duration of the quiet eye depending on the type of task (e.g., Williams et al., 2002; Vickers, 2007). In the specific case of golf putting, experts’ quiet eye period lasted in between 2 and 3 s, while non-experts’ quiet eye durations lasted around 1.5 s (e.g., Vickers, 1992, 2007). Furthermore, performance has been shown to be directly related to quiet eye duration. For example, in golf longer quiet eye periods have been reported for successful putts compared to unsuccessful putts (e.g., Wilson and Pearcy, 2009; Vine et al., 2013). Overall, the quiet eye has been found to be a major factor related to perceptual-cognitive expertise, differentiating between experts and non-experts (e.g., Mann et al., 2007).

Although the underlying mechanisms of the quiet eye are still highly debated (for overviews, see Klostermann, 2014; Gonzalez et al., 2015), the most prominent account assumes that the quiet eye behavior relates to a critical period of information processing related to the motor action to be executed (e.g., Vickers, 1996, 2009). As such, the quiet eye suggests that higher order cognitive processes control gaze behavior (e.g., Williams et al., 2002). This behavior should be based on prior experience, and thus on the degree of elaborateness of the mental representation (for details, see next paragraph). In other words, the more experienced the individual, the more elaborate the representation is, and the more functional the information processing directly relating to the motor action should be. In this sense, mental representation development and prolongation of the quiet eye should relate to one another. However, despite the tremendous amount of research on expert-novice differences with respect to quiet eye behavior, research examining the changes in quiet eye behavior over the course of learning is lacking. Both the quiet eye and its changes over time as well as its relation to the underlying mental representation, have yet to be examined.

Perceptual-cognitive approaches to motor control and learning assume motor actions to be guided by way of representations containing information about the perceptual effects of the actions (e.g., theory of anticipative behavioral control: Hoffmann, 1993; theory of event coding: Hommel et al., 2001; cognitive action architecture approach: Schack, 2002, 2004). According to the cognitive action architecture approach (CAA-A; for an overview, see Schack and Ritter, 2009), motor actions are represented in memory as well-integrated representational networks or taxonomies comprised of perceptual-cognitive units (i.e., basic action concepts; BACs). Analogous to object representations and the idea of basic object concepts (e.g., Rosch and Mervis, 1975; Rosch, 1978; Mervis and Rosch, 1981; Hoffmann, 1986, 1990), BACs represent cognitive compilations of movement elements/body postures and their corresponding perceptual effects, which are closely tied to the attainment of action goals (e.g., Schack, 2004, 2012). For instance, grip check as a BAC of the golf putt is thought to represent a cognitive chunk serving a particular action goal (i.e., to ensure an optimal grip during the preparation of the putting movement before initiation of the backswing). As such it is comprised of the corresponding body posture (e.g., standing up right, hips flexed, upper body leaning forward, holding the putter in hands) and movement elements (e.g., take grip, move fingers until in right position) together with their sensory consequences (e.g., feel hands touching the surface of club; sense slight pressure in fingers, see both hands touch each other; for other BACs related to putting, see **Table 1**).

**Table 1:** Basic action concepts (BACs) of the golf putt.

*N*°	Basic action concept (BAC)	Movement phase
(1)	Shoulders parallel to target line	Preparation
(2)	Align club face square to target line	
(3)	Grip check	
(4)	Look to the hole	

(5)	Rotate shoulders away from the ball	Backswing
(6)	Keep arms-shoulder triangle	
(7)	Smooth transition	

(8)	Rotate shoulders toward the ball	Forward swing
(9)	Accelerate club	

(10)	Impact with the ball	Impact
(11)	Club face square to target line at impact	
(12)	Follow-through	
(13)	Rotate shoulders through the ball	

(14)	Decelerate club	Attenuation
(15)	Direct clubhead to planned position	
(16)	Look to the outcome	


*Each of the 16 basic action concepts (BACs) of the golf putt can be functionally assigned to one of the movement phases. The numbers on the left relate to the different BACs; they do not reflect a particular order, but serve to better display the concepts in **Figures 3**–**5.***

Mental representations of complex action are believed to consist of hierarchical taxonomies comprised of BACs. The arrangement and clustering of these BACs within the taxonomy are important, because they control and guide the execution of the skill. Motor learning, according to the CAA-A, is reflected by functional changes in the arrangement and organization of BACs within the taxonomies that are held within long-term memory. Specifically, during learning by repeated (imagined or actual) execution of a complex action, the relations and the groupings of action concepts (i.e., mental representation structure) are modified (e.g., Schack, 2003, 2004, 2006; Schack and Ritter, 2013). This process is suggested to result in action-related structure formation (i.e., perceptual-cognitive scaffolding) in long-term memory.

Until now, experts have been shown to differ significantly from novices in the way these representations are structured in long-term memory, with experts holding structured representations with groupings of BACs reflecting the functional phases of the motor action, whereas the representations of non-experts were not meaningfully or functionally organized (e.g., Schack and Mechsner, 2006; Bläsing et al., 2009; Stöckel et al., 2015). More recently, the mental representation of a motor action has been shown to functionally adapt in the direction of an elaborate representation during motor learning, thereby relating more so to the biomechanical task demands (Frank et al., 2013, 2014; Land et al., 2014). Specifically, Frank et al. (2013) investigated the changes in skill representation during motor learning incorporating physical practice. The authors found that skill representation of novices practicing (i.e., repeatedly executing without technical instructions) a golf putting task for several days changed over the course of practice such that the novices’ representations developed in the direction of that of an expert. More specifically, novices’ unstructured representations became more structured over time, with the groupings of BACs pertaining to the movement phases of the putt (i.e., the preparation, the forward swing and the impact) after practice. In contrast, novices who did not practice the putt revealed no changes in their underlying representation structure, and thus their representation remained unstructured.

In a more recent study, the adaptation of mental representation according to type of practice was investigated (Frank et al., 2014). Novices practiced the golf putt under one of four conditions: mental practice, physical practice, combined mental, and physical practice, and no practice. Both putting performance and mental representation of the putt were assessed prior to and after 3 days of practice, and again after a 72 h retention period. While the putting performance of the groups reflected improvements as expected (i.e., the combined practice group performing best, followed by the physical practice group, while the mental practice group performed worst after practice), mental representations developed differently between the groups. While the physical practice group showed only marginal changes in representation structure over time, both the mental practice and the combined practice group revealed major changes in their representations of the putt after the acquisition phase. That is, after mental practice and after combined mental and physical practice, the trained novices elicited more elaborate representation structures, reflecting the functional phases of the movement (i.e., the preparation phase, the swing and the impact phase, and the attenuation phase). From this, representation structures seem to develop differently during motor learning, depending on the type of practice.

In sum, although much attention has been directed toward differences between experts and novices with respect to their perceptual-cognitive background (e.g., mental representations, quiet eye behavior), less research has focused on the development and change of these perceptual-cognitive components over the course of motor learning. From expert-novice differences alone, no clear conclusions can be made regarding the type and extent of motor and perceptual-cognitive changes that occur during the learning process. Thus, it is essential to take a longitudinal perspective, in order to learn about the perceptual and cognitive changes that occur within the motor action system over the course of this process and to be able to effectively guide the motor learning process. Accordingly, the main purpose of the present study was to investigate the influence of practice (i.e., physical practice and combined physical plus mental practice) on both the mental representation and quiet eye behavior of learners practicing a golf putting task. Furthermore, we were interested in whether the structure formation in mental representation related to the duration of the quiet eye after learning.

In line with previous research, we expected putting performance to improve as a result of practice, with the combined physical plus mental practice group performing equivalent or better than the physical practice only group after practice with regards to motor performance. Regarding perceptual-cognitive changes, we expected mental representations to develop over the course of practice, with representation structures being more elaborate after practice compared to no practice. In addition, we expected combined physical plus mental practice to reveal more elaborate representations compared to physical practice only. Furthermore, given that mental representations underpin the processing of task related information, longer quiet eye durations were expected to be associated with more developed mental representations. Consequently, we predicted that quiet eye durations would be longer following practice in comparison to no practice. Finally, we expected quiet eye durations to be longest for the type of practice revealing the most elaborate representations after practice (i.e., the combined physical plus mental practice).

## Materials and Methods

### Participants

Forty-five university students participated in the present study. None of the participants had any prior experience with golf putting. Participants were assigned to one of three conditions^1^: combined mental and physical practice (*n* = 16, *M*_age_ = 24.38 years, *SD* = 2.73, 8 female), physical practice (*n* = 15, *M*_age_ = 25.73 years, *SD* = 2.99, 10 female) and no practice (*n* = 14, *M*_age_ = 27.00 years, *SD* = 8.74, 9 female). The study was conducted in accordance with local ethical guidelines, and conformed to the declaration of Helsinki.

### Tasks and Measures

#### Outcome Performance

Participants performed a golf-putting task on an artificial indoor putting green (size: 4 m × 9 m), using a standard putter and a standard golf ball. The task consisted of putting the ball to a target three meters away from the starting point. The target, projected onto the surface of the green via an overhead projector, corresponded to the size of a regulation golf hole (i.e., 10.8 cm in diameter). Participants were asked to putt the golf ball as accurately as possible to the target, on which the ball was supposed to stop. Putting performance was recorded by way of a motion capture system (Vicon Motion Systems, Oxford, UK). Specifically, 6 T10 CCD cameras captured and tracked the ball rolling and stopping. The recordings were made with a temporal resolution of 200 Hz and a spatial resolution of approximately 0.25 mm.

#### Mental Representation Structure

Structural dimensional analysis of mental representation (SDA-M) was employed to assess mental representation structures of the putt, providing psychometric data on the structuring and dimensioning of mental representations of complex movements in long-term memory (for more details, see Schack, 2012). In other words, the SDA-M serves to determine relations between and the grouping of basic action concepts (i.e., BACs) of a motor action. For the specific purpose of the present study, a pre-determined set of 16 BACs of the putt were used (see **Table 1**), each BAC pertaining to one particular movement phase of the golf putt: preparation (BAC 1–4), backswing (BAC 5–7), forward swing (BAC 8–9), impact (10–13), and attenuation (BAC 14–16).

The SDA-M consists of several steps. In a first step, a split procedure (for more details, see next paragraph) is performed resulting in a distance scaling between the BACs of a predetermined set. Next, a hierarchical cluster analysis is used to outline the structure of the given set of BACs. Following this, a factor analysis can be used in order to determine the dimensions in the structured set of BACs. In a last step, an analysis of invariance within- and between-groups serves to compare different cluster solutions (for details, see Schack, 2012). In addition, similarity between cluster solutions was examined (for details on both analyses, see Data Analysis section).

More specifically, the splitting task (i.e., first step of the SDA-M) proceeds as follows: while one BAC of the putt is permanently shown on a computer screen (i.e., the anchor concept), the rest of the BACs are displayed one after another in randomized order. For each of the BACs being displayed together with the anchor concept, participants are asked to decide whether the given BAC is related to the anchor concept or not during movement execution. Once the participant has finished a list of BACs, another BAC takes the anchor position and the procedure continues. After each BAC has been compared to the remaining BACs (*n*-1), the splitting task is completed.

#### Gaze Behavior

Gaze behavior was measured by way of eye-tracking during putting. Accordingly, eye-movements were recorded using a head-mounted portable eye-tracking system with an eye and a scene camera. Specifically, the SMI iViewX HED mobile eye-tracker is a corneal reflex system that operates monocular at a sampling rate of 200 Hz, with a gaze position accuracy <0.5°-1°. Each recorded scene video had a resolution of 376 pixels × 240 pixels at 25 fps (1 frame = 40 ms). This system allows for the recording of eye-movements in natural environments in which participants move and interact with their environment while performing complex movements (i.e., vision in action paradigm; see Vickers, 2007).

#### Imagery Ability

To assess visual and kinesthetic imagery ability, the revised version of the Movement Imagery Questionnaire (MIQ-R; Hall and Martin, 1997) was administered. During this procedure, participants perform, then imagine and finally rate their imagery experience of four different movements. During their imagery, participants are instructed to either “see” or “feel” one of the four movements without actually performing. Following this, participants are asked to rate the ease or difficulty of imaging the movement on a 7-point Likert scale. Thus, participants imagine each of the four movements, by focusing either on the visual modality or the kinesthetic modality separately by instruction, resulting in a final rating of eight items.

#### Manipulation Check

In order to control whether participants performed the imagery as instructed (cf. Goginsky and Collins, 1996), a post-experimental questionnaire was administered after each practice session to participants practicing mentally. Specifically, we asked participants of the combined mental and physical practice group to report their imagery in detail. First, participants were asked to describe the imagery content shorthand. Second, participants had to rate on a 7-point Likert scales (1 = *very difficult*, 7 = *very easy*; 1 = *never*, 7 = *always*), how easy it had been to follow the instructions in general, how often they used an external perspective and how often they used an internal perspective. Third, participants were asked to indicate how easy it had been to “see” and how easy it was to “feel” the putt during their imagery. Finally, we asked participants whether they had experienced any problems and, if so, to describe them in detail.

### Procedure

The present study consisted of a pre-test, an acquisition phase of three consecutive days of practice, a post-test, and a retention-test after 3 days of rest (see **Table 2**).

**Table 2:** Design of the study including three test days and an acquisition phase of 3 days.

	Pre-test	Acquisition			Post-test	Retention-test
				
	Day 1	Day 2	Day 3	Day 4	Day 5	Day 8
Combined physical and mental practice (CP) group (*n* = 16)	Eye-tracking	Physical + mental practice *(executed and imagined putts)*			Eye-tracking	Eye-tracking


	Putting task				Putting task	Putting task
	SDA-M				SDA-M	SDA-M

Physical practice (PP) group (*n* = 15)	Eye-tracking	Physical practice *(executed putts only)*			Eye-tracking	Eye-tracking


	Putting task				Putting task	Putting task
	SDA-M				SDA-M	SDA-M

No practice (NP) control group (*n* = 14)	Eye-tracking	No practice *(neither executed nor imagined putts)*			Eye-tracking	Eye-tracking


	Putting task				Putting task	Putting task
	SDA-M				SDA-M	SDA-M


*SDA-M: structural dimensional analysis of mental representation; putting task on test days: 2 warm- up putts followed by 20 putts; putting practice during acquisition phase: 3 × 20 imagined and executed putts (combined mental and physical practice group) or 3 × 10 executed putts (physical practice group) per day for the practice groups.*

#### Pre-test

At the beginning of the study, participants signed informed consent forms. Next, in order to become familiar with the task at hand, participants watched a video showing a skilled golfer performing the putting task. Following this, the eye-tracking system was calibrated, employing a standard five-point calibration procedure. In order to assess participants’ initial putting performance and gaze behavior, each participant performed two warm-up putts followed by 20 putts. Participants were asked to putt a golf ball as accurately as possible to the target, on which the ball was supposed to stop. After the putting, an introduction to the splitting task was given. This procedure served to assess the participant’s initial mental representation structure of the putt. Accordingly, in order to ensure comprehension of the concepts, a randomized list of the 16 BACs of the putt was presented and explained to the participants. After having read general instructions on how to complete the splitting task, participants were explicitly instructed to decide on a yes/no-basis whether the presented basic action concepts were related to one another or not during movement execution. Following this, participants performed the splitting task. Finally, each participant completed the MIQ-R as an indicator of imagery ability.

#### Acquisition Phase

During the next 3 days, participants either practiced the putt (practice groups), or did not partake in putting practice (control group).

##### Physical practice (PP) group

Three blocks of 10 putts were performed on each practice day in the PP condition. Prior to each block, participants were asked to putt as accurately as possible to the target, on which the ball was supposed to stop. Importantly, no other information than the visible outcome of the putt (i.e., knowledge of result) was available to the participants. That is, no additional information such as technical feedback (i.e., knowledge of performance) was given to the participants during the acquisition phase.

##### Combined mental and physical practice (CP) group

Three blocks of 20 putts were performed on each practice day in the combined mental and physical practice condition, with each block consisting of 10 imagined and 10 actual putts. Prior to each block, participants were asked to take the starting position. While participants were standing upright on the green with the putter in their hands, the imagery script was read out loud to each participant. Participants were asked to imagine the putting movement as well as the ball rolling toward the target and stopping on the target, as predefined by the script. They were further told to imagine from an internal perspective, to incorporate all the senses in their imagery, and to try and imagine as clearly and as vividly as possible. The information on imagery perspective, imagery modality, and imagery vividness was intentionally given in order to control for as many aspects during imagery as possible and to optimize the efficacy of the imagery intervention (cf. Holmes and Collins, 2001). As soon as the reading was finished, participants imagined repeatedly the putting movement on their own. Participants were asked to hold their eyes closed during their imagery and to slightly raise their index finger each time they had finished a putt in their minds. This procedure allowed the participant to concentrate on themselves and their imagery and, at the same time, to make it possible for the experimenter to control for the number of imagined putts per block without disturbing the participants’ imagery. Next, during actual putting, participants were instructed to putt as accurately as possible to the target, on which the ball was supposed to stop. No technical instructions were given. Finally, participants filled out a post-experimental questionnaire at the end of each practice session.

##### No practice (NP; control) group

During the acquisition phase, the control group neither imagined nor executed the putt.

#### Post- and Retention-Test

Participants were retested 1 day after the acquisition phase, as well as after a retention interval of 3 days. Prior to post-test and retention-test assessment, the same standard five-point calibration procedure was used to calibrate the eye-tracking system. Next, each participant performed again the two warm-up putts followed by 20 putts. Both their gaze behavior and putting performance were measured. Following this, all participants completed the splitting task in order to determine their final mental representation structures of the putting movement.

### Data Analysis

#### Outcome Performance

By capturing the final ball position after each putt, putting performance was assessed. From these data, two outcome variables were calculated for each test day. Specifically, based on the *x* and *y* coordinates of each putt with the center of the target as origin of the axes, two-dimensional error scores were determined (for details, see Hancock et al., 1995). Accordingly, putting accuracy was measured by mean radial error (MRE). MRE is defined as a subject’s average distance of each putt outcome from the center of the target in mm. Putting consistency was measured by bivariate variable error (BVE), analogous to variable error in one-dimensional analyses. BVE was defined as the square root of a subject’s k shots’ mean squared distance from their centroids in mm. A subject’s centroid is a positionally typical shot whose coordinates are given by the average *x* and average *y* value of a subject’s shots in mm.

In order to ensure that performance between groups did not differ at pre-test, a 1 × 3 [test day (pre) × group (PP, CP, NP) ANOVA was performed on both MRE and BVE. For putting performance over time, a 3 × 3 test day (pre, post, retention) × group (PP, CP, NP)] mixed-model ANOVA with test day as a within-subjects factor and group as a between-subjects factor was performed on each of the dependent variables. For *post hoc* analysis, independent *t*-tests were conducted. A Holm-Bonferroni correction was employed in order to account for the inflation of type I errors (Holm, 1979). Cohen’s *d* was used as an estimate of effect size (Cohen, 1992).

#### Mental Representation Structure

The structure of each participant’s mental representation was determined by way of a cluster analysis. With the help of this procedure, the information on the distances between BACs, as obtained by the splitting task, was transformed into dendrograms outlining the structure of the BACs of the putt. For the purpose of the present study, mean group dendrograms were calculated for each group and test day (for more details see Schack, 2012). An alpha-level of α = 0.05 was chosen for all cluster analyses, resulting in a critical value *d*_crit_ = 3.41. To explain, BACs in a given cluster solution were considered not related when being linked above this critical value, while BACs were considered related when being linked below this value and thus resulted in a cluster.

To compare cluster solutions, two analyses were conducted. First, analyses of invariance were used to learn about differences between cluster solutions (Lander, 1991, 1992; see Schack, 2012). Accordingly, cluster solutions are considered different (i.e., variant) for α < 0.68, while cluster solutions are considered the same (i.e., invariant) for α ≥ 0.68. Second, to further examine the similarity between cluster solutions and a reference, the adjusted rand index (ARI; Rand, 1971; Santos and Embrechts, 2009) was used. The ARI serves as an index of similarity, ranging on a scale from -1 to 1. Indices between “-1” and “1” mark the degree of similarity between two cluster solutions, with “1” indicating that two cluster solutions are the same. For the purpose of the present study, ARI was used to investigate the degree of similarity between mean group dendrograms and an expert dendrogram reflecting well the movement phases (i.e., preparation, backswing, forward swing, impact, and attenuation).

#### Gaze Behavior

The quiet eye period was assessed by the duration of the final fixation before movement onset for each putt (see Vickers, 2007, 2009). Accordingly, eye-tracking data were analyzed frame by frame. The number of frames for the final fixation prior to the initiation of the backswing was coded. From this, fixation duration for each putt was calculated. In line with previous studies (e.g., Vine and Wilson, 2010), the quiet eye analysis was performed on a subset of trials (i.e., every fourth); a total of 675 putts. Furthermore, due to problems during the tracking of one participant’s eye movements resulting in poor data quality, the data of one subject were excluded from subsequent data analyses. In order to ensure that groups did not differ in their quiet eye behavior at pre-test, a 1 × 3 [test day (pre) × group (PP, CP, NP)] ANOVA was performed on quiet eye duration.

In order to examine the quiet eye over time, a 3 × 3 [test day (pre, post, retention) × group (PP, CP, NP)] mixed model ANOVA with test day as within subjects factor and group as between subjects factor was performed on final fixation duration prior to movement onset. For *post hoc* analyses, independent *t*-tests were conducted. Again, a Holm-Bonferroni correction was employed in order to account for the inflation of type I errors (Holm, 1979) and Cohen’s *d* was used as an estimate of effect size (Cohen, 1992).

Finally, in order to investigate the relationship between elaborateness in mental representation structure and gaze behavior after learning, a one-tailed Pearson product-moment correlation was computed between adjusted rand indices for each individual’s representation structure (in comparison to the expert reference structure) and quiet eye duration.

## Results

### Imagery Ability

Participants in the CP group scored 44.38 (*SD* = 6.07; 5.55 per item) on average for overall imagery ability, 23.63 (*SD* = 2.66; 5.91 per item) for visual imagery ability, and 20.75 (*SD* = 5.00; 5.12 per item) for kinesthetic imagery ability. Thus, participants’ average score per item was approximately 6, *easy to see*, for the visual imagery ability scale, and 5, *somewhat easy to feel*, for the kinesthetic imagery ability scale. This is considered as being sufficient for subsequent mental practice (e.g., Smith and Collins, 2004; Smith et al., 2008).

### Manipulation Check

For the CP group, participants’ manipulation check responses were analyzed to control whether participants adhered to the instructions given during mental practice sessions. With respect to imagery content, each participant mentioned in their imagery descriptions both the putting movement and the ball rolling, indicating that they had imagined the content that had been instructed. For the internal imagery perspective, mean scores during acquisition phase were 6.29 (*SD* = 0.69), *very often*, and for external imagery perspective 2.36 (*SD* = 1.42), *rarely*. Thus, participants of the CP group performed their imagery mainly from an internal perspective. In addition, participants found it easy to “see” and to “feel” the movement while imaging. Specifically, participants scored an average of 5.47 (*SD* = 0.96), *somewhat easy to see*, for visual imagery and 4.78 (*SD* = 1.33), *somewhat easy to feel*, for kinesthetic imagery. Moreover, participants in general found it easy to follow the instructions during imagery, as indicated by mean scores of 5.52 (*SD* = 1.04). Also, none of the participants reported any problems during imagery sessions. From this, it can be assumed that participants of the CP group had been able to perform the imagery as instructed. This was considered a prerequisite for subsequent data analyses.

### Outcome Performance

**FIGURE 1:**
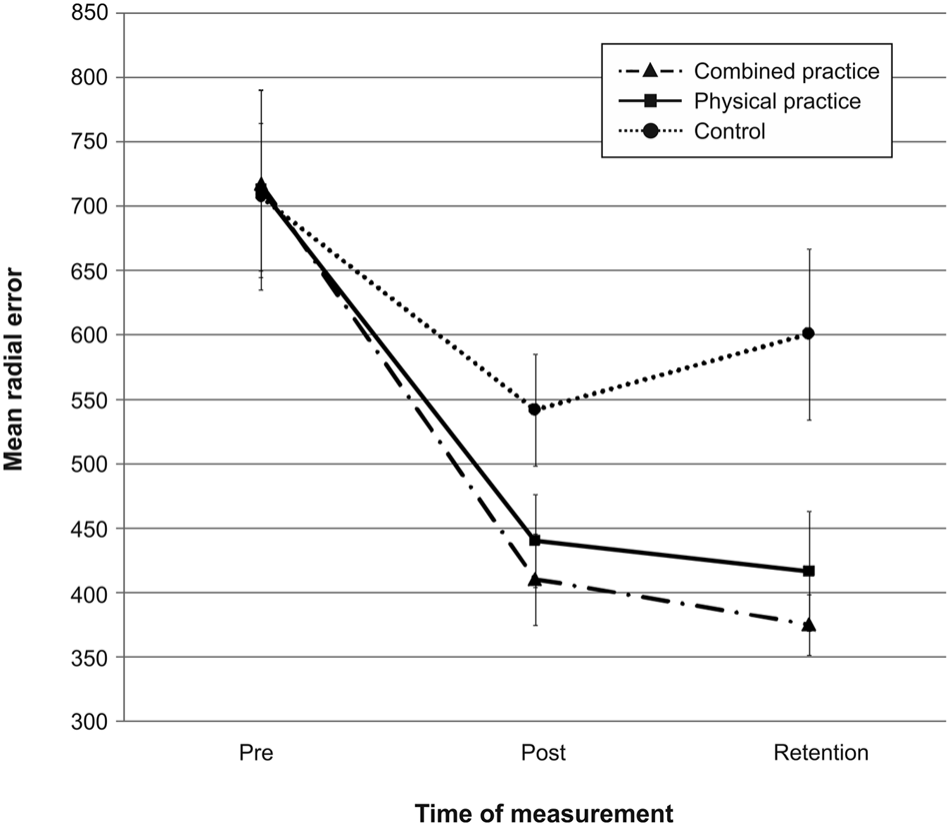
**Mean radial error (i.e., accuracy) in mm from pre-test to post-, and retention-test.** The different lines relate to the different conditions (i.e., no practice, physical practice or combined mental and physical practice). Error bars represent standard errors.

**FIGURE 2:**
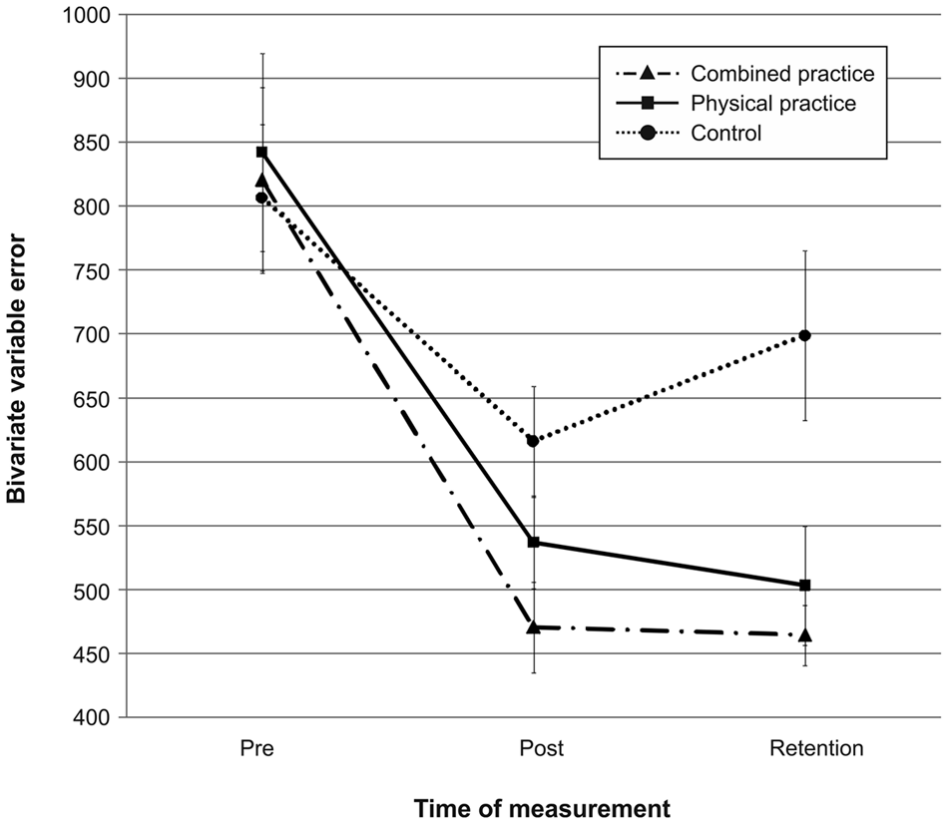
**Bivariate variable error (i.e., consistency) in mm from pre-test to post-, and retention-test.** The different lines relate to the different conditions (i.e., no practice, physical practice or combined mental and physical practice). Error bars represent standard errors.

Mean radial error and bivariate variable error of the three groups from pre-, to post-, and to retention-test is displayed in **Figures 1** and **2.** In addition, descriptives are presented in **Table 3.** Both, MRE *F*(2,42) = 0.008, *p* = 0.992, $\eta_{p}^{2}$ = 0.000, and BVE *F*(2,42) = 0.086, *p* = 0.918, $\eta_{p}^{2}$ = 0.004, did not differ between groups at pre-test. For accuracy, a repeated measures ANOVA on MRE indicated a significant *test day* × *group* interaction, *F*(4,84) = 4.042, *p* = 0.005, $\eta_{p}^{2}$ = 0.161. *Post hoc* analyses revealed that only the CP group, *t*(28) = -2.893, *p* = 0.007 (α_crit_ = 0.017), *d* = 1.05, putted significantly more accurate than the NP group at post-test. The difference between the PP group and the NP group failed to reach significance, *t*(27) = -2.167, *p* = 0.039 (α_crit_ = 0.025), *d* = 0.80. Furthermore, no significant differences were found between the PP and the CP group, *t*(29) = -0.740, *p* = 0.465 (α_crit_ = 0.05), *d* = 0.27. After a retention-interval of 3 days, however, both the CP group, *t*(15.830) = -3.813, *p* = 0.002 (α_crit_ = 0.017), *d* = 1.43, and the PP group, *t*(27) = -2.748, *p* = 0.011 (α_crit_ = 0.025), *d* = 1.01, performed with greater putting accuracy compared to the NP group, while no difference was found between the PP and the CP group, *t*(29) = -0.998, *p* = 0.327 (α_crit_ = 0.05), *d* = 0.35. For consistency, a repeated measures ANOVA on BVE indicated a significant *test day* × *group* interaction, *F*(4,84) = 3.615, *p* = 0.009, $\eta_{p}^{2}$ = 0.147. *Post hoc* analyses revealed that the CP group putted more consistently compared to the NP group at post-test, *t*(22.815) = -2.989, *p* = 0.007 (α_crit_ = 0.017), *d* = 1.11, while this was not the case for the PP group, *t*(27) = -1.343, *p* = 0.191 (α_crit_ = 0.025), *d* = 0.50. Furthermore, the CP and the PP group did not differ in their putting consistency, *t*(29) = -1.361, *p* = 0.184 (α_crit_ = 0.05), *d* = 0.49. For retention-test, both the CP group, *t*(18.590) = -3.299, *p* = 0.004 (α_crit_ = 0.017), *d* = 1.23, and the PP group, *t*(27) = -2.534, *p* = 0.017 (α_crit_ = 0.025), *d* = 0.94, performed with greater consistency in comparison to the NP group, whereas the PP and the CP group did not differ, *t*(29) = -0.732, *p* = 0.470 (α_crit_ = 0.05), *d* = 0.26.

**Table 3:** Descriptive statistics for performance outcome variables across pre-test, post-test, and retention-test for each of the groups in cm.

	Pre-test		Post-test		Retention-test	
			
Group	*MRE M (SD)*	*BVE M (SD)*	*MRE M (SD)*	*BVE M (SD)*	*MRE M (SD)*	*BVE M (SD)*
NP (*n* = 14)	70.75 (18.10)	80.68 (17.43)	54.18 (13.73)	61.58 (15.26)	60.07 (21.03)	69.88 (24.07)
PP (*n* = 15)	71.31 (24.53)	84.23 (27.10)	44.02 (11.48)	53.71 (16.26)	41.64 (14.75)	50.31 (17.18)
CP (*n* = 16)	71.76 (22.96)	82.06 (23.99)	41.01 (11.20)	47.04 (10.62)	37.50 (7.43)	46.43 (12.09)


*NP, no practice; PP, physical practice; CP, combined (mental and physical) practice; MRE, mean radial error (accuracy); BVE, bivariate variable error (consistency).*

In sum, while only the combined practice led to more accurate and more consistent putting after the acquisition phase, both types of practice proved superior in improving putting accuracy and consistency compared to no practice after a 3 day retention interval (see also **Figures 1** and **2**).

### Mental Representation Structure

**FIGURE 3:**
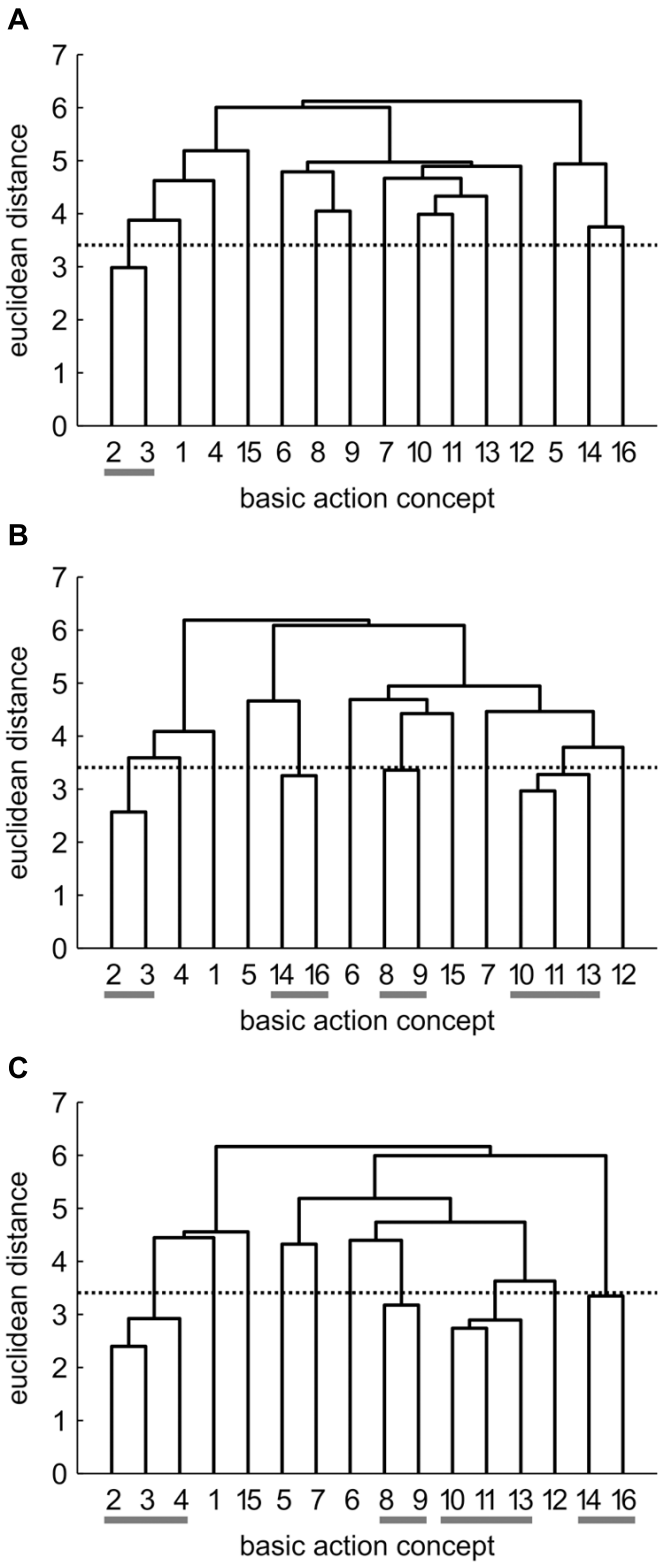
**Mean group dendrograms of the combined mental and physical practice group (*n* = 16) for the golf putt at **(A)** pre-test, **(B)** post-test, and **(C)** retention-test.** The numbers on the *x*-axis relate to the BAC number, the numbers on the *y*-axis display Euclidean distances. The lower the link between related BACs, the lower is the Euclidean distance. The horizontal dotted line marks *d*_crit_ for a given α-level (*d*_crit_ = 3.41; α = 0.05): links between BACs above this line are considered not related; horizontal gray lines on the bottom mark clusters. BACs: (1) shoulders parallel to target line, (2) align club face square to target line, (3) grip check, (4) look to the hole, (5) rotate shoulders away from the ball, (6) keep arms-shoulder triangle, (7) smooth transition, (8) rotate shoulders toward the ball, (9) accelerate club, (10) impact with the ball, (11) club face square to target line at impact, (12) follow-through, (13) rotate shoulders through the ball, (14) decelerate club, (15) direct club head to planned position, and (16) look to the outcome.

**FIGURE 4:**
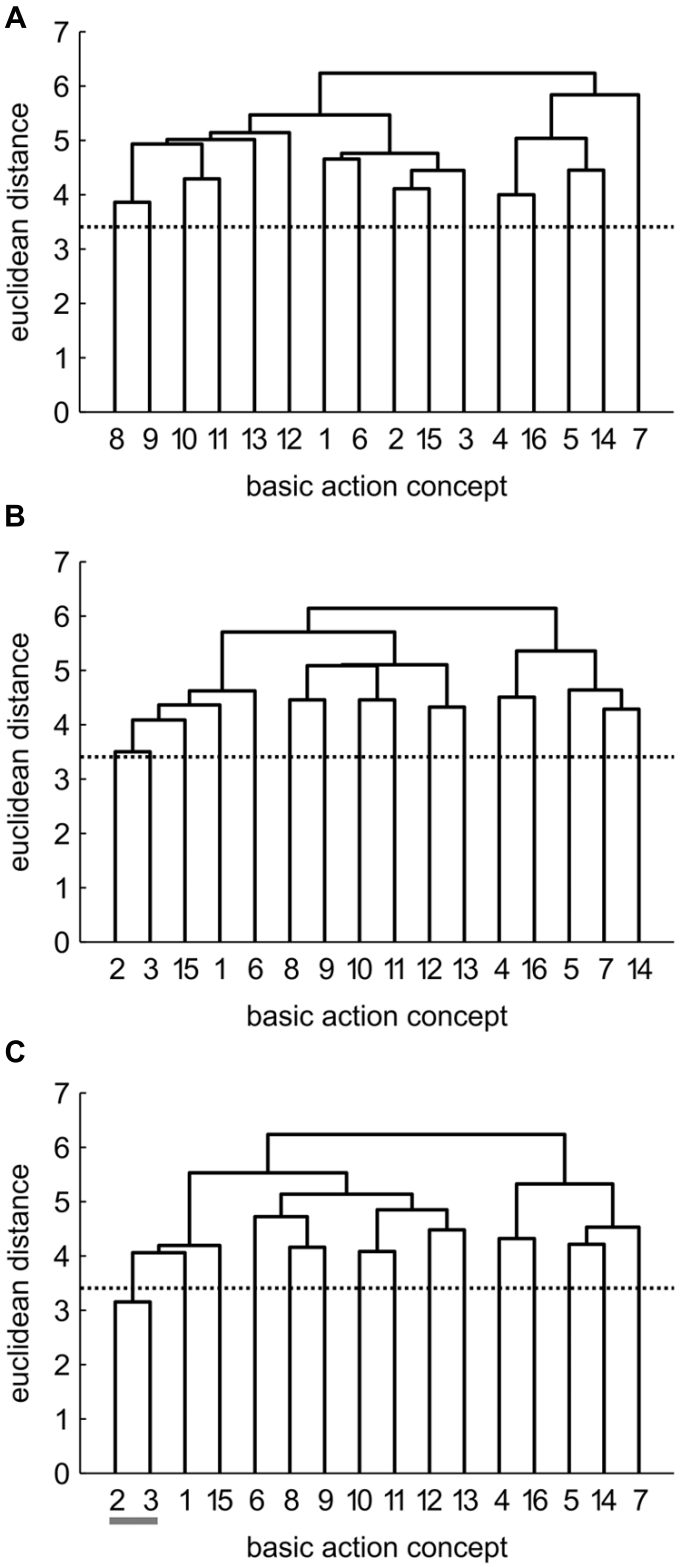
**Mean group dendrograms of the physical practice group (*n* = 15) for the golf putt at **(A)** pre-test, **(B)** post-test, and **(C)** retention-test (α = 0.05; *d*_**crit**_ = 3.41)**.

**FIGURE 5:**
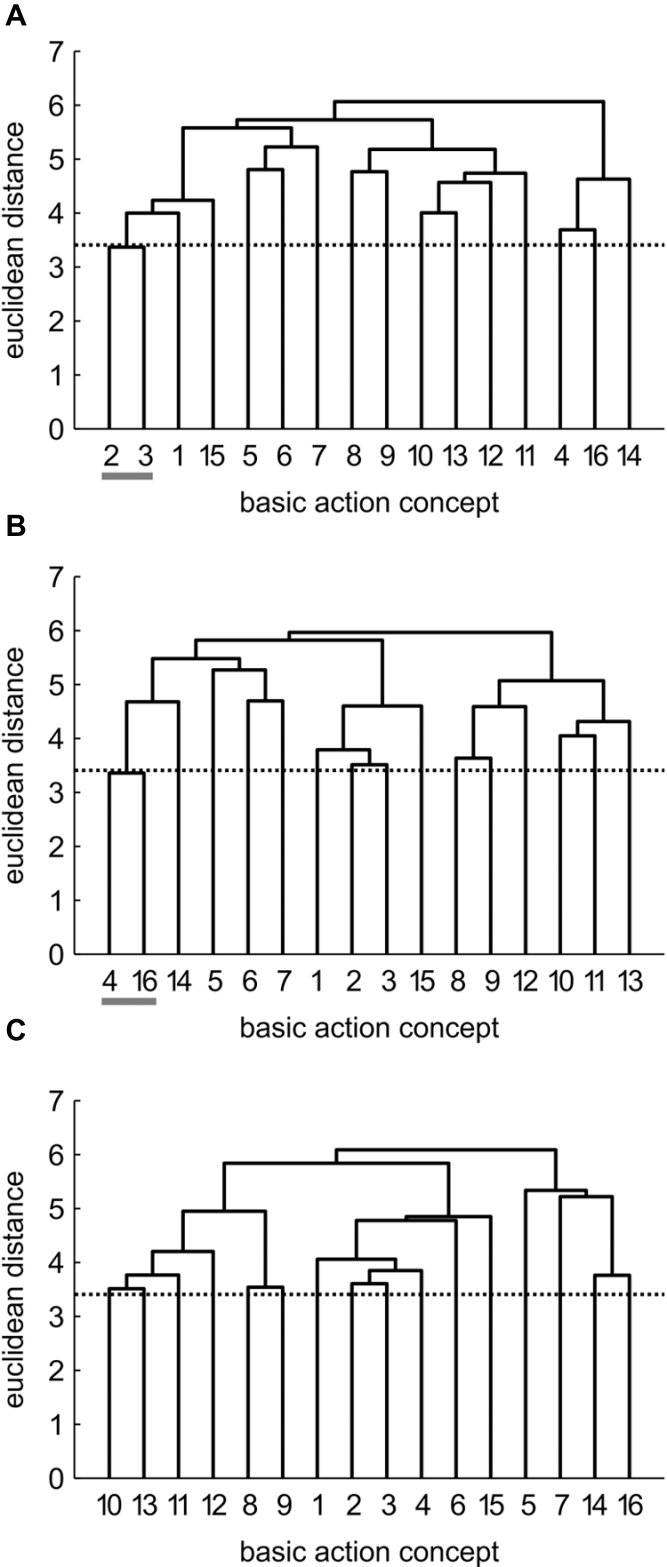
**Mean group dendrograms of the no practice control group (*n* = 14) for the golf putt at **(A)** pre-test, **(B)** post-test, and **(C)** retention-test (α = 0.05; *d*_crit_ = 3.41)**.

Mean group dendrograms of the three groups from pre-, to post- and to retention-test are displayed in **Figures 3**–**5.**

#### Combined Mental and Physical Practice (CP) Group

As seen in **Figure 3**, no structure was evident for the CP group prior to the acquisition phase. In detail, the mean group dendrogram of the CP group revealed only one cluster pertaining to the preparation phase (BAC 2 and 3). After the acquisition phase, however, the mean group dendrogram was comprised of four clusters relating to different phases of the movement [i.e., preparation phase (BAC 2, 3), forward swing (BAC 8, 9), impact (BAC 10, 11, 13), and attenuation (BAC 14, 16)]. Cluster solutions of the CP group for post- and retention-test were similar, with the only difference being that after the 3 days retention interval, the cluster pertaining to preparation phase involved one more concept (BAC 2, 3, 4). Thus, for the CP group, the number of functional clusters increased over the course of acquisition phase, with the representation structure becoming more elaborate over time. The descriptive changes over time observed in the dendrograms were confirmed by analyses of invariance. Specifically, while the cluster solutions for pre- and post-test (λ = 0.24) as well as for pre- and retention-test (λ = 0.24) were variant (i.e., significant changes in the structures over practice), the two cluster solutions of post- and retention-test (λ = 0.71) were invariant (i.e., no meaningful differences between representation structures). Furthermore, increases in adjusted rand indices from pre-test (*ARI*_pre_ = 0.12) to post-test (*ARI*_post_ = 0.35) and to retention-test (*ARI*_retention_ = 0.50) indicate increasing similarity in comparison to an expert structure and as such emphasize that the changes in representation structure reflect a functional development.

#### Physical Practice (PP) Group

The mean group dendrograms of the PP group revealed minimal clustering over time (see **Figure 4**). Specifically, while no clustering was evident for the PP group prior and after the acquisition phase, the retention test revealed one cluster pertaining to preparation phase (BAC 2 and 3). As there was no overlap in the clustering of the different cluster solutions, analysis of invariance resulted in values of 0. More important, increasing adjusted rand indices over time (*ARI*_pre_ = 0, *ARI*_post_ = 0, *ARI*_retention_ = 0.12) suggest a minimal development in direction of the expert representation structure.

#### No Practice (NP) Group

Prior to the acquisition phase, the mean group dendrogram of the NP group revealed one cluster relating to the preparation of the putting movement (BAC 2 and 3; see **Figure 5**). After the acquisition phase, however, a different structure emerged which reflected one cluster comprised of two functionally unrelated concepts (BAC 4 and 16). Although being related in the sense that both concepts involve the word “look,” these concepts are not related during movement execution. Thus, this relation is based on superficial rather than on functional characteristics. After the 3 days retention interval, no clusters were evident in the mean group dendrogram of the NP group. Analysis of invariance resulted in values of 0, as there was no overlap in the clustering of the different cluster solutions. When being compared to the expert structure, ARIs even revealed a slight decrease in the degree of similarity from pre-test (*ARI*_pre_ = 0.12) to post-test (*ARI*_post_ = -0.02) to retention-test (*ARI*_retention_ = 0.00).

In sum, prior to the acquisition phase, mental representations revealed little to no structures. Over the course of practice, however, combined practice led to a significant development in mental representation structure, while physical practice only led to little functional changes in mental representation structure compared to no practice (see also **Figures 3–5**).

### Gaze Behavior

**FIGURE 6:**
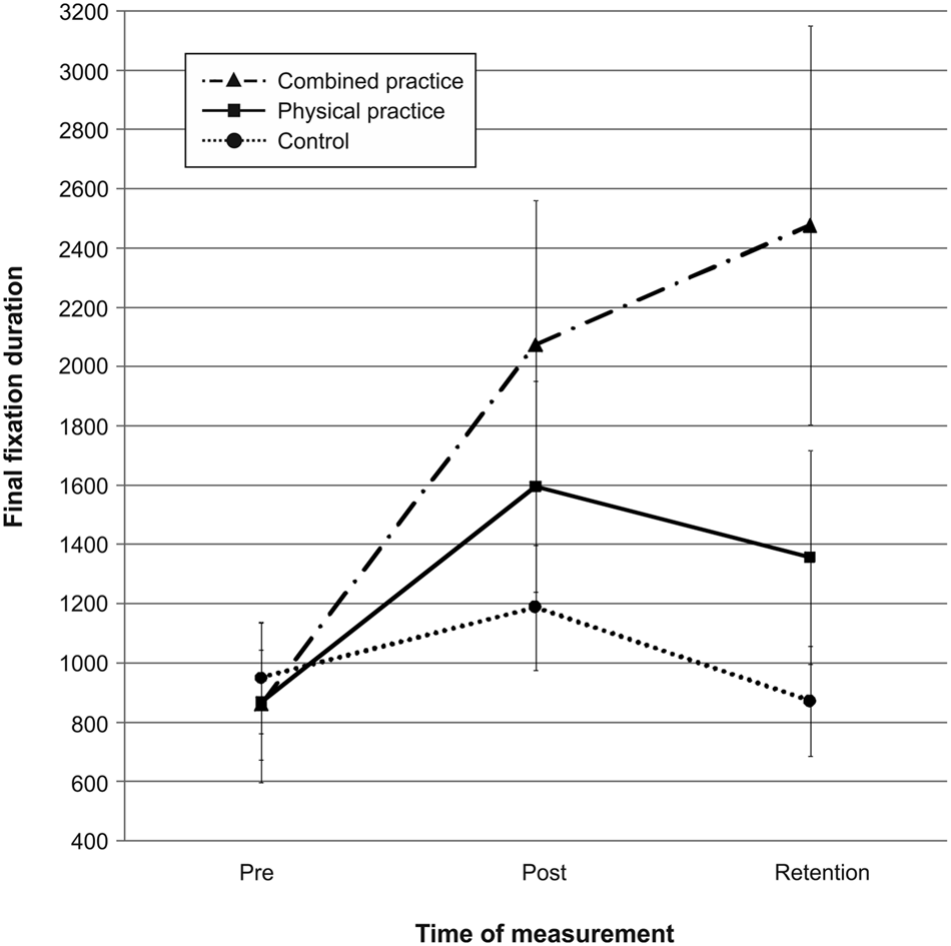
**Mean quiet eye duration in ms from pre-test to post- and retention-test.** The different lines relate to the different conditions (i.e., no practice, physical practice or combined mental and physical practice). Error bars represent standard errors.

Mean quiet eye durations for the three groups across pre-, post-, and retention-test are presented in **Figure 6.** Quiet eye duration did not differ between groups at pre-test, *F*(2,41) = 0.076, *p* = 0.927, $\eta_{p}^{2}$ = 0.004. A repeated measures ANOVA on quiet eye duration indicated a significant *test day* × *group* interaction, *F*(4,82) = 6.532, *p* < 0.001, $\eta_{p}^{2}$ = 0.242. However, *post hoc* analyses revealed that neither the CP group, *t*(20.928) = 2.079, *p* = 0.050 (α_crit_ = 0.017), *d* = 0.74, nor the PP group, *t*(26) = 1.167, *p* = 0.254 (α_crit_ = 0.025), *d* = 0.44, demonstrated longer quiet eye durations compared to the NP group after 3 days of practice. Furthermore, the CP group and the PP group did not differ in their quiet eye duration after acquisition phase, *t*(28) = 0.954, *p* = 0.348 (α_crit_ = 0.05), *d* = 0.35. After 3 days of rest, however, the CP group demonstrated significantly longer quiet eye durations compared to the NP group, *t*(17.563) = 2.887, *p* = 0.010 (α_crit_ = 0.017), *d* = 1.03. Again, quiet eye durations of the PP group were not different from those of the NP group, *t*(26) = 1.418, *p* = 0.168 (α_crit_ = 0.050), *d* = 0.54. The difference in quiet eye duration between the CP group and the PP group failed to reach significance, *t*(28) = 1.753, *p* = 0.090 (α_crit_ = 0.025), *d* = 0.65.

In sum, after 3 days of rest, the duration of the quiet eye was longer for the combined practice compared to no practice, while this was not the case for physical practice. Interestingly, after the retention interval, a tendency was becoming evident that the combined practice had led to even longer quiet eye durations than the physical practice. Thus, combined mental and physical practice as opposed to physical practice only led to a longer quiet eye period during the acquisition of putting skill in comparison to no practice in the present study (see also **Figure 6**).

Pertaining to the relation between gaze behavior and mental representation structure, a Pearson product-moment correlation between quiet eye durations and individual ARIs revealed a small positive correlation between the two variables, *r* = 0.286, *n* = 44, *p* = 0.030, with better developed representation structures relating to longer quiet eye durations after learning. In other words, participants with the most elaborate mental representation structure after learning also revealed the longest duration in quiet eye behavior.

## Discussion

The purpose of the present study was to examine the influence of practice on the mental representation of a golf putt, gaze behavior prior to putting (i.e., the quiet eye), and putting performance. By doing so, we aimed at gaining further insights into the perceptual-cognitive background of performance changes that occur during the process of learning a motor action. Specifically, we were interested in whether (overt) motor changes (i.e., changes in putting accuracy and consistency) would be realized in (covert) cognitive and perceptual changes (i.e., changes in mental representation structure and quiet eye duration) as a result of two different practice conditions (i.e., physical practice and physical plus mental practice in comparison to no practice).

Regarding the representation of the golf putt in long-term memory, novices’ mental representation structures developed over the course of practice, with the combined physical plus mental practice leading to the most elaborate representation structures relative to an expert structure. Physical practice alone led to fewer changes in direction of an expert structure. No such changes in representation structures were observed in the control condition. The results of the present study are in line with previous research on differences in mental representation of complex action according to skill level, with well-experienced athletes revealing more structured representations than their less-experienced counterparts (e.g., Schack and Mechsner, 2006; Bläsing et al., 2009; Velentzas et al., 2010). Moreover, the present findings on the development of mental representation structures over time further support the idea that the learning of a motor action is associated with functional organization of action-related knowledge in long-term memory (e.g., Frank et al., 2013, 2014; Land et al., 2014). As expected, representation structures developed most during combined physical plus mental practice, while during physical practice only, less development was evident. Representation structures of the group that incorporated mental practice into their practice regimen revealed several functional clusters of BACs in the present study. Clusters were functional in the sense that they pertained to the functional movement phases of the putt (i.e., preparation, forward swing/impact, attenuation) after a retention interval of 3 days. For the group practicing only physically, mental representation structure revealed fewer, but some clustering at retention-test, while no clustering was evident for the group not practicing at all. Thus, the results of the present study confirm the findings by Frank et al. (2013, 2014) such that when spending time practicing, the mental representation structure of a complex motor action develops functionally (i.e., becomes more similar to the representation of an expert).

A main aim of the present study was to further investigate the perceptual-cognitive background of performance changes by having a closer look at the quiet eye and its changes over the course of learning. In the present study, the combined physical plus mental practice led to longer quiet eye periods (i.e., longer fixation durations prior to putting) compared to no practice. This finding fits well into the body of research on the quiet eye, which has repeatedly shown longer quiet eye durations for higher-skilled athletes in comparison to lower-skilled athletes (e.g., Vickers, 1992, 1996; for an overview, see Vickers, 2007).

More importantly, our results extend findings on differences in quiet eye behavior between skilled and unskilled individuals by providing insight into the change in quiet eye behavior over the course of practice. First, to our knowledge, the present study is the first to show that quiet eye duration changes in novices practicing a complex motor action. Specifically, the present findings show that practice is associated with a longer quiet eye period. Second, the quiet eye developed alongside of mental representations in the present study, with mental representation structures and quiet eye durations relating to one another after learning. This indicates that the more elaborate information-processing during movement preparation, as expressed by longer quiet eye durations, is based on more elaborate underlying mental representations in long-term memory. As such, the results of the present study further support the notion that the quiet eye is rooted in the cognitive domain (e.g., Williams et al., 2002; Klostermann et al., 2014; Gonzalez et al., 2015), reflecting critical action-related information processing that is based on the representation available. This might be indicative of quiet eye duration reflecting a predicitive mode of control initiating a cognitively demanding process of motor planning. In order to address this in more detail, future studies are planned to investigate the link between structure formation in long-term memory, capacity in short-term memory (i.e., chunking^2^) and their relationship to the quiet eye, as they relate to the learning of a motor action.

Regarding performance changes over time, outcome performance improved over the course of practice in the present study, with both types of practice leading to improved accuracy and consistency. Improvements in performance persisted over 3 days of no practice, thus reflecting stable changes. In this sense, and according to the traditional view of motor learning, both practice conditions (i.e., physical practice and physical practice plus mental practice) led to motor learning (e.g., Magill, 2011; Schmidt and Lee, 2011). Relatively permanent changes in putting performance as a result of practice as found in the present study are in line with the general idea that repeatedly executing a motor action leads to improved performance of that motor action (e.g., Newell and Rosenbloom, 1981).

Interestingly, additional mental practice did not lead to superior putting performance in the present study. One reason why additional mental practice might not have contributed to superior overt putting performance in the present study is the smaller relative magnitude of effect that mental practice has in comparison to physical practice. Meta-analyses investigating the relative magnitude of effect between mental and physical practice emphasize the superiority of physical practice over mental practice (e.g., Feltz et al., 1988; Driskell et al., 1994). For instance, Driskell et al. (1994) reported strong effect sizes for physical practice (*d* = 0.78) and moderate effect sizes for mental practice (*d* = 0.53). Hence, the smaller magnitude of effect may be one reason why additional mental practice in the present study did not prove effective in further enhancing motor performance and supporting motor learning on the motor output level. As a consequence, extending the practice phase (i.e., amount of sessions and/ or amount of trials) may elicit a distinct effect of additional mental practice.

Related to that, a second plausible explanation for the lack of differences between physical plus mental practice and physical practice only may be that mental practice effects may not primarily become evident in terms of overt performance during early skill acquisition. Focusing on motor performance, Driskell et al. (1994) had a closer look at mental practice effects relative to level of expertise (i.e., novice vs. experienced individuals) in their meta-analysis. In novices, mental practice was found to be more beneficial for cognitive tasks in comparison to motor tasks, while experienced individuals profited both in cognitive and motor tasks (between-task comparison). More recently, findings reported by Frank et al. (2014) suggested that mental practice particularly promotes the cognitive level of action organization in novices practicing a motor action (within-task comparison). Specifically, the authors examined the development of mental representation structure over the course of learning in novices, practicing either by imagery, by execution, by a combination of both, or did not practice at all. From this study, mental practice has shown to particularly promote representation structure development, resulting in more elaborate representation structures for the groups involving mental practice. This suggests that mental practice particularly affects the perceptual-cognitive level at an early stage of motor learning. Accordingly, these changes do not necessarily have to transfer one-to-one to the motor output level, and thus do not necessarily or only minimally have to be reflected in overt outcome performance.

In the present study, combined physical plus mental practice contributed to both more elaborate representation structures of the putt and longer quiet eye durations before initiation of the putting movement as well as improved putting performance in comparison to the control group. In contrast, physical practice led to improved putting performance, but to fewer changes in representation structures and shorter quiet eye durations. Moreover, in comparison to the physical practice condition, combined practice particularly promoted perceptual-cognitive changes within the motor action system. First, representation structures of the combined group were more similar to an expert structure compared to the ones of the physical practice group. Second, while not statistically significant, the difference between the two practice groups after the retention interval indicated a medium effect size (*d* = 0.65), with the combined practice leading to longer fixation durations. From these findings, combined practice seems to have influenced the motor system differently than physical practice only, with combined physical plus mental practice influencing the motor action system more so on a perceptual-cognitive level.

This might be indicative of a differential influence of mental practice (i.e., repeated motor imagery) and physical practice (i.e., repeated motor execution) with regards to different levels within the motor action system (e.g., Frank et al., 2014). While the principle of functional equivalence (Finke, 1979; Johnson, 1980; Jeannerod, 1994, 1995) and the simulation theory (Jeannerod, 2001, 2006) postulate that the imagery and the execution of an action are functionally equivalent, as both states to some degree involve the activation of the motor action system, this principle does not specify the (similar or differential) influence that each of these states of action has on the motor action system during learning (for related discussions, see e.g., Munzert et al., 2009; Guillot et al., 2013; Wakefield et al., 2013). In other words, the unique potential of mental practice and physical practice to induce changes on different levels within the motor action system during learning remains to be specified. Using a four group design (mental practice, physical practice, mental and physical practice, no practice), Frank et al. (2014) investigated the influence of mental and physical practice on mental representation development in novices. After the same amount of practice, the groups practicing by way of motor imagery (either solely or in combination with motor execution), revealed more functional representation structures after learning, while no differences were evident in overt motor performance between the practice groups after learning. A similar pattern (i.e., no differences in overt motor performance, but differences in covert perceptual-cognitive variables) was found in the present study, which might be interpreted as further evidence that mental practice operates primarily on higher levels within the motor action system during early skill acquisition (for a more detailed discussion, see Frank, 2014; Frank et al., 2014).

It is important to note, however, that the amount of practice trials differed between the two practice groups in the present study, with the combined group practicing mentally in addition to the physical practice. Therefore, an alternative explanation for the differences between practice groups in the present study is the difference in amount of practice. Specifically, the combined physical plus mental practice group practiced twice the amount of trials (30 executed plus 30 imagined putts per practice session) than did the physical practice group (30 executed putts per practice session) in the present study. Thus, any difference found between the two practice groups might be solely attributed to the difference in amount of practice and not to mental practice itself. Future research is needed in order to investigate the differential effect of physical and mental practice on the perceptual-cognitive components of motor action, and the quiet eye in particular.

To conclude, the present study shed light on the perceptual-cognitive background of performance changes during motor learning. By doing so, it was possible to gain further insights relating to three research areas: (1) as for quiet eye research, we were able to show by way of a longitudinal design that the quiet eye becomes longer in novices practicing a motor action, and that quiet eye durations relate to the degree of development in underlying representation structures; (2) as for motor imagery research, this study was the first to investigate motor learning incorporating motor imagery with a focus on the quiet eye as a window into action-related information processes; and (3) as for motor learning research in general, we demonstrated the value of looking at motor skill acquisition from different angles, considering both overt changes in motor performance and covert perceptual-cognitive changes which take place within the motor action system during learning. Taking a multifaceted view in future studies may contribute to bringing forward research on some of the remaining unanswered questions in our fields.

## Author Contributions

Conception and design of study (CF, WL, TS); Data collection (CF); Analysis and interpretation of data (CF, WL, TS); Drafting of manuscript (CF, WL, TS).

## Conflict of Interest Statement

The authors declare that the research was conducted in the absence of any commercial or financial relationships that could be construed as a potential conflict of interest.

## Footnotes

1 Participants were assigned to the different groups based on their initial putting performance at pre-test. No significant differences existed between the groups at the initiation of putting performance.

2 We thank the reviewer for his/her comments related to this point.
